# Effects of aeolian deposition on soil properties and microbial carbon metabolism function in farmland of Songnen Plain, China

**DOI:** 10.1038/s41598-024-65578-0

**Published:** 2024-06-26

**Authors:** Jixian Mo, Ziwei Song, Yanjing Che, Jie Li, Tianyi Liu, Jingyi Feng, Ziying Wang, Jiandong Rong, Siyu Gu

**Affiliations:** 1 College of Resources and Environment, Northeast Agricultural University, Harbin, 150030 China; 2 College of Life Science and Agriculture and Forestry, Qiqihar University, Qiqihar, 161006 China; 3 Qiqihar Experimental Station, Heilongjiang Province Hydraulic Research Institute, Qiqihar, 161006 China

**Keywords:** Aeolian deposition, Soil properties, Metagenomics, Carbon metabolism, Songnen Plain, Environmental impact, Soil microbiology, Sedimentology

## Abstract

The effects of wind erosion, one of the crucial causes of soil desertification in the world, on the terrestrial ecosystem are well known. However, ecosystem responses regarding soil microbial carbon metabolism to sand deposition caused by wind erosion, a crucial driver of biogeochemical cycles, remain largely unclear. In this study, we collected soil samples from typical aeolian deposition farmland in the Songnen Plain of China to evaluate the effects of sand deposition on soil properties, microbial communities, and carbon metabolism function. We also determined the reads number of carbon metabolism-related genes by high-throughput sequencing technologies and evaluated the association between sand deposition and them. The results showed that long-term sand deposition resulted in soil infertile, roughness, and dryness. The impacts of sand deposition on topsoil were more severe than on deep soil. The diversity of soil microbial communities was significantly reduced due to sand deposition. The relative abundances of Nitrobacteraceae, Burkholderiaceae, and Rhodanobacteraceae belonging to α-Proteobacteria significantly decreased, while the relative abundances of Streptomycetaceae and Geodermatophilaceae belonging to Actinobacteria increased. The results of the metagenomic analysis showed that the gene abundances of carbohydrate metabolism and carbohydrate-activity enzyme (GH and CBM) significantly decreased with the increase of sand deposition amount. The changes in soil microbial community structure and carbon metabolism decreased soil carbon emissions and carbon cycling in aeolian deposition farmland, which may be the essential reasons for land degradation in aeolian deposition farmland.

## Introduction

The loss of soil nutrients and the coarsening of soil texture caused by wind erosion, leading to soil desertification, is one of the critical reasons for soil degradation in arid and semi-arid regions worldwide^1^. Over one hundred countries worldwide, approximately 88.67 × 10^7^ km^2^ of land, were affected by wind erosion, accounting for 59.55% of the global land area^2^. In northern China, most arid and semi-arid grasslands and farmlands were affected by wind erosion. Approximately 6.84 × 10^5^ km^2^ of farmland was buried by moving dunes^3^. Wind erosion transported much sand to neighboring or further areas downwind when wind eroded farmland and caused degradation^4,5^. The migrated sand was mixed into the soil through sedimentation and infiltration, resulting in desertification in the downwind farmland^6,7^. Bioaerosols in the atmosphere carried various microorganisms, such as bacteria, fungi, and viruses, which play an essential role in global climate change by influencing physical and chemical processes in the atmosphere^8^. Bioaerosols were generated at the land/atmosphere interface and transported over long distances by sand and dust events in the downwind environment, affecting the soil microbial community structure and ecosystem in the settling area^9,10^. Sand sediment contained more sand and fewer nutrients, which coarsened soil texture, decreased water holding capacity, and increased soil temperature, seriously damaging agricultural productivity^11^. Sand deposition has been identified as a serious threat to sustainable crop production^12^.

The Songnen Plain is one of the largest grain production bases in China. Its western region is semi-arid, with an annual precipitation of 350–450 mm, making it susceptible to wind erosion^6^. In recent decades, wind erosion has rapidly expanded from Horqin Sandy Land and Hulunbeier Sandy Land to the Black Soil Belt^13^. Desertification caused by sand deposition has become the leading cause of soil degradation in farmland. The rise in global temperature, excessive cultivation, and misuse of land have led to this spread^1,14^. At present, 7.50% of the Songnen Plain area, approximately 5.96 × 10^4^ km^2^ of farmland^15^, is affected by wind erosion and sand deposition, with an annual increase of 0.44%^16^. It has become the fifth most extensive sandy land in China—Songnen Sandy Land. Severe sandy desertification made the ecosystem of the Songnen Plain more susceptible to erosion. However, few studies evaluated the effect of sand deposition on farmland and ecosystems in the Songnen Plain.

Soil carbon cycling is a vital surface system process closely related to global climate change, and any changes in soil microorganisms are one of the intrinsic driving factors for global climate change^17^. Wind erosion significantly negatively affects soil microbial biomass and community structure in farmland, weakening the network complexity^18^ and metabolic function of bacterial communities^19^. The reduction of soil nutrients caused by sand accumulation may have a similar effect on soil microorganisms in aeolian deposition farmland. Research on grassland showed that sand deposition significantly increased soil microbial α-diversity and the abundance of Actinobacteria and Cyanobacteria^20,21^. At the same time, functional genes related to carbon cycling, including carbon fixation and degradation^21^, were significantly reduced, which seriously interfered with the carbon metabolism of the soil, further exacerbating soil degradation. However, due to the difficulty of simulating the wind erosion process and highly complex agricultural ecosystems^22^, the effect of sand accumulation on soil microbial communities and carbon metabolism in farmland is still unclear.

This study collected soil samples from typical sand deposition farmland with different amounts of sand accumulation in the Songnen Plain of China. The effects of sand deposition on soil properties and microbial carbon metabolism function were analyzed after measuring soil respiration, soil properties, and soil microbial and carbon metabolism gene abundances. In addition, we attempted to explain how microorganisms regulated species composition and carbon metabolism functions to adapt to adverse environments such as soil desertification, barrenness, and drought. Our research aims were (1) to elucidate that sand deposition was an essential driving factor for soil degradation in farmland, (2) to identify the critical microbial species that affected soil carbon metabolism in sand deposition farmland, and (3) to clarify that the changes in soil microbial communities and carbon metabolism were the fundamental reasons for the degradation of sand deposition farmland.

## Materials and methods

### Study area

**Figure 1:**
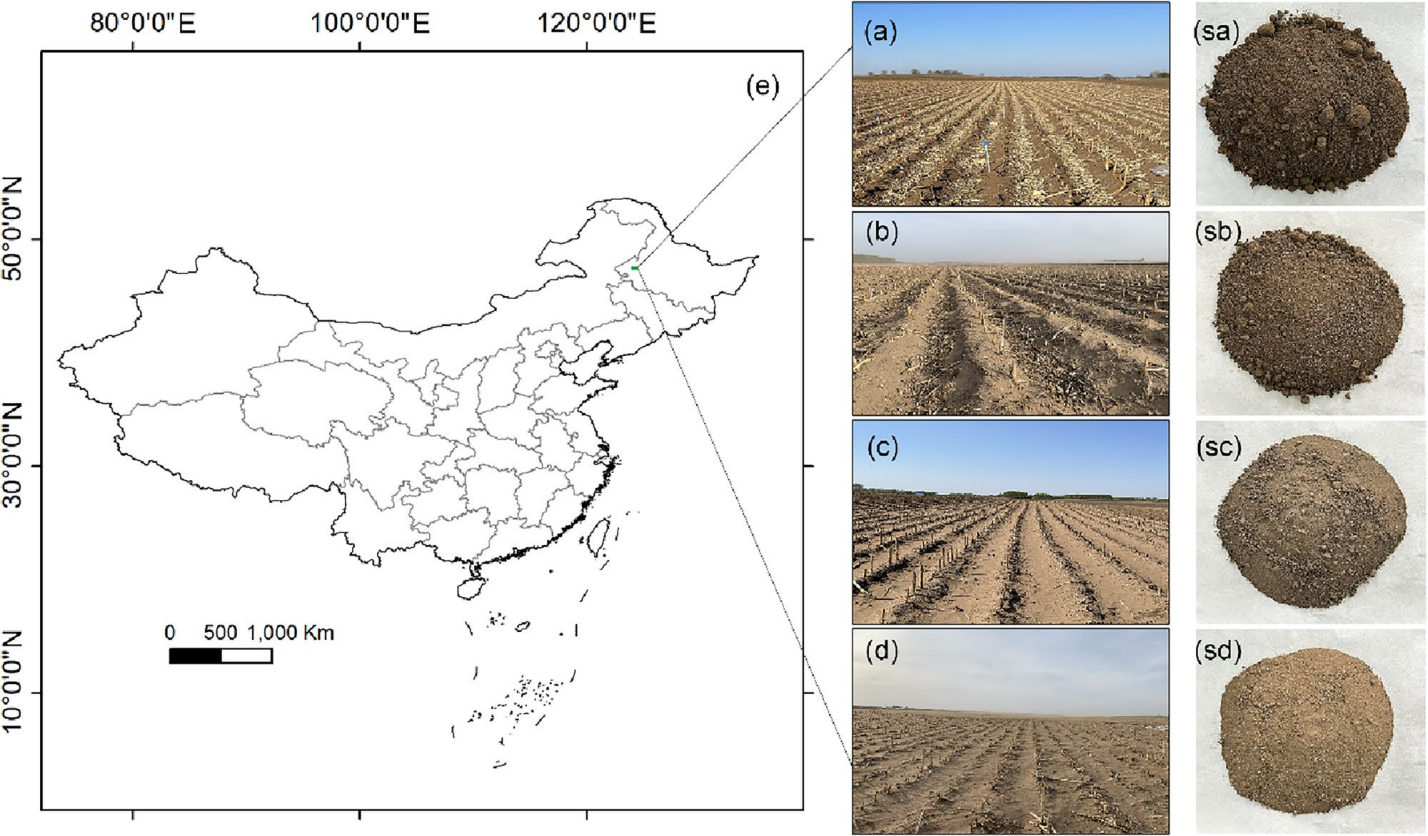
Location map of the study site in the Songnen Plain, China (**e**). The landscapes with typical aeolian deposition of the shallow-sand deposit (SSD)(**b**), the moderate-sand deposit (MSD)(**c**), and the deep-sand deposit (DSD)(**d**), and the landscape of the control farmland with non-sand deposit (CNSD)(**a**). Sa, sb, sc and sd were soil samples collected from four farmlands, respectively.

The sampling area is located in the farmland of Maris Dowoer District in the northern of Songnen Plain, China (123° 56′–123° 59′ E, 47° 36′–47° 39′ N, elevation 154–158 m) (Fig. 1). This area has a semi-arid low-temperature continental-monsoon climate, with an annual rainfall of 350–450 mm and an annual average temperature of 3.2 °C^23^. The rainfall from June to August accounts for over 70% of the annual rainfall^24^. The annual potential evapotranspiration (PET) exceeds 1000 mm, and the ratio of yearly precipitation to PET is as low as 0.2–0.5^25^. This area is a typical wind erosion area^13^, with a soil layer thickness of about 30–45 cm in farmland, mainly planted with corn.

In this study, the cultivation method of farmland was ridge tillage with the same management methods. Corn was usually treated with stubble after harvest, with a stubble height of 10–30 cm. According to the International Soil Texture Classification Standard, the soil in the study area belongs to sandy soil and sandy loam. Layer A contains 0.32–1.51% organic carbon, 0.04–0.57% clay, 67.09–92.63% sand, and a pH of 5.34–5.72 (Table S1 and S2). This system planted corn in late May and harvested in October each year. The wind erosion was pronounced in spring (from March to May). From the thawing of frozen topsoil in mid-March to the drying and exposure of loose topsoil in early May, farmland soil was highly susceptible to wind erosion^26^.

### Experimental design

This study was conducted during the year 2023. Based on the on-site investigation, a broad and flat wind erosion farmland plot (2000 × 500 m) was selected as the research object (Fig. 1e). This area is part of the long-term monitoring station operated by the Qiqihar Wind Erosion Monitoring and Research Center of the Ministry of Water Resources of China. The annual cultivation mixed the deposited sand with the original surface soil. Field observation indicated that the west side of the farmland was the windward zone, with significantly higher wind speeds than the east side. The sand accumulation intensity in farmland showed signs of increasing to varying degrees during the wind erosion season, with a marked gradient from east to west. Four sample belts were established along the gradient, each with a length and width of 100 × 10 m and a distance of 500 m between each sample belt. Each sample belt was divided into three 10 × 10 m sampling quadrats as three replicates with an interval of 30 m. Several rulers were randomly erected in each sampling quadrat to measure the thickness of sand deposition in farmland during the wind erosion season from March to May each year. Based on soil bulk density and average sand deposition thickness, the sand deposition amount (SDA) and sand deposition rate (SDR) of each sampling quadrat were calculated using the following formula:

1 $$ {\text{SDA}} = {\text{ADT}} \times {\text{BD}} \times {1}0 $$

where SDA is the sand deposition amount of soil sampling quadrat in farmland (kg/m^2^), ADT is the average deposition thickness (cm), and BD is the soil bulk density (g/cm^3^).

2 $$ {\text{SDR}} = {\text{SDA}}/t $$

where SDR is the sand deposition rate of the soil sampling quadrat (kg/m^2^·d), and *t* is the time during measuring sand deposition (d).

The results are shown in Table S1. The amounts of sand deposition in the four farmland belts were 31.07–33.6, 13.99–19.45, 7.35–9.79, and 0.01–0.35 kg/m^2^, respectively. The four farmland belts were divided into four groups according to the amounts of sand deposition: deep-sand deposit (DSD) (Fig. 1d and sd), moderate-sand deposit (MSD) (Fig. 1c and sc), shallow-sand deposit (SSD) (Fig. 1b and sb), and non-sand deposit (CNSD) (Fig. 1a and sa).

### Soil sampling and analysis

**Figure 2:**
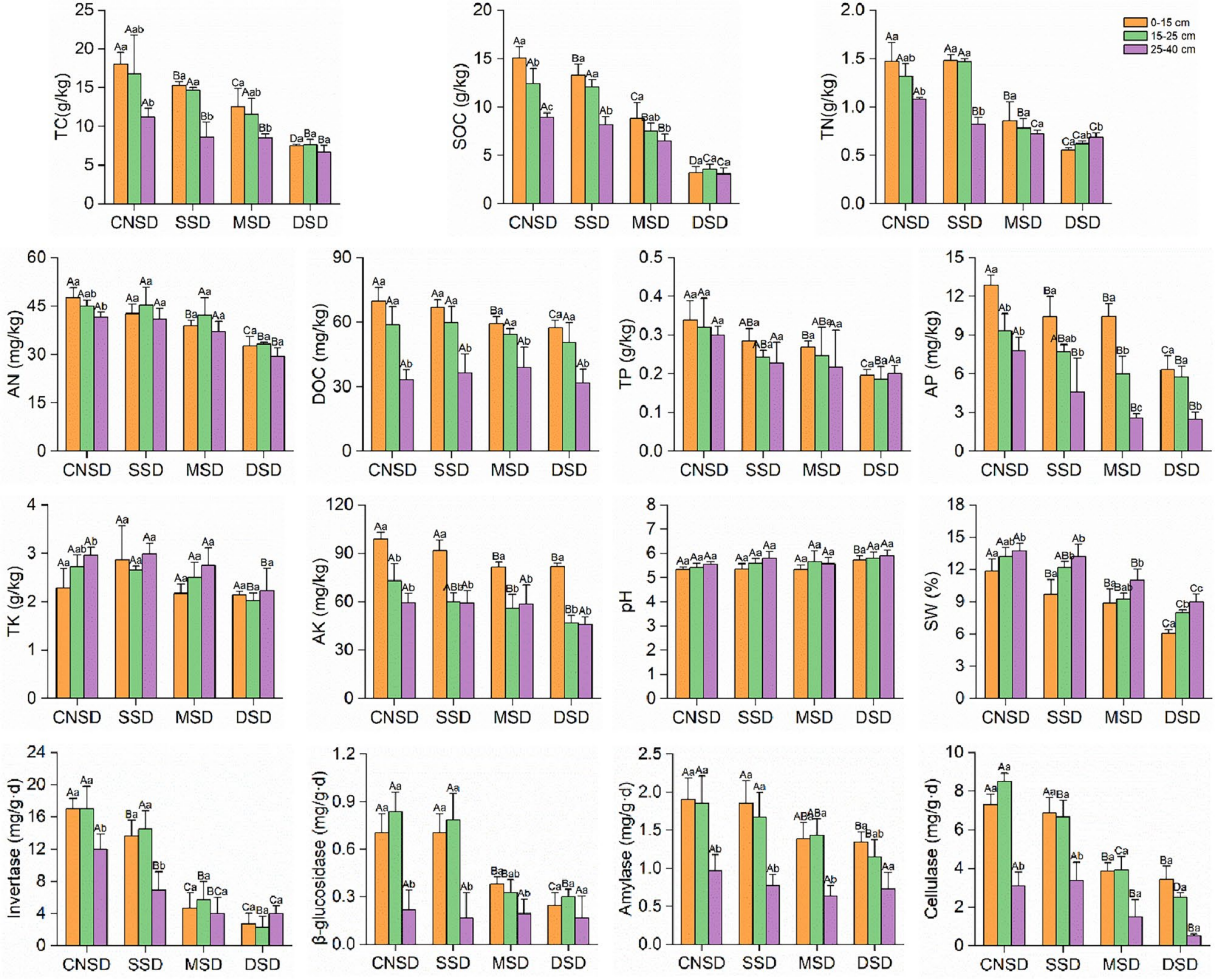
The soil physicochemical properties in the aeolian deposition farmland. Bars with different capital letters indicated significant differences at the same depth in the different sand deposition farmlands (*P* < 0.05). Bars with different lowercase letters indicated significant differences at different depths in the same sand deposition farmland (*P* < 0.05). Every value was expressed as the mean ± SD (n = 3).

The sampling time was selected in mid-May before ploughing and sowing. In each quadrat, soil samples were collected from 0 to 15, 15 to 25, and 25 to 40 cm using the five-point sampling method respectively and each layer was mixed into a composite sample. Three composite samples were collected within each sample belt as three replicates. The soil samples were placed in sealed plastic bags and transported to the laboratory. Each sample was thoroughly sieved to 2 mm in the laboratory to remove roots and incorporate litter. Then, each of them was divided into three sub-samples: one was air-dried at room temperature for soil physicochemical properties analysis, another was kept fresh and incubated in the laboratory for soil CO_2_ emissions measurement, and the third was immediately stored in a freezer at − 80 °C for microbiological analysis. Soil texture was determined by a laser particle size analyzer (LS-609, OMEC, China) and identified using the International Society of Soil Science (ISSS) soil texture classification system. The total C (TC) and total N (TN) were measured by Elemental Analyzer (EA3000, EuroVector, Italy), total K (TK) by Flame Photometer (FP6410A, Jingke, China) after alkali melting, and total P (TP) by UV–Vis Spectrophotometer (752N, INESA, China) after H_2_SO_4_-HCIO_4_ digestion. Soil organic carbon (SOC) and dissolved organic carbon (DOC) were measured using the K_2_Cr_2_O_7_–H_2_SO_4_ oxidation method. Soil available N (AN) was measured by the alkaline diffusion method, available P (AP) by the Olsen method, and available K (AK) by Flame Photometer after ammonium acetate extraction. The soil water content was measured using the drying method. The activities of soil invertase, amylase, and cellulase were measured using the 3,5-dinitrosalicylic acid colorimetric, and the activity of soil β-glucosidase was measured using *p*-nitrophenol colorimetric. The soil properties are shown in Table S1 and Fig. 2.

### Soil CO_2_ emissions measurement

Twelve fresh topsoil samples (4 soil sample belts × 3 replicates) were sieved through a 2 mm sieve to remove stones and plant residues. 100 g dry weight equivalent of each soil sample was placed into a 1000 ml wide-mouthed jar with a spiral cap and pre-cultured at 25 °C for 5 days. The moisture content of the soil samples was adjusted with deionized water to maintain their initial state during the pre-cultured period. A 25 ml beaker containing 5 ml of 1 M NaOH was placed at the bottom of each jar. Then, the jars were sealed and incubated in the dark at the same temperature for 24 h to trap CO_2_. In addition, three bottles without soil samples were used as blanks to eliminate the CO_2_ from the air inside the jars. The soil CO_2_ emissions (μg CO_2_/g soil/day) were estimated by titrating 5 ml of each trap and 5 ml 1 M BaCl_2_ (1:1) with 0.1 M HCl and phenolphthalein indicator (1% w/v in ethanol) according to Butterly et al.^27,28^.

### Soil microbial biomass carbon measurement

Soil microbial biomass carbon (SMBC) was measured using the Chloroform Fumigation Extraction (CFE) technique described by Anshumali and was appropriately modified^29^. 10 g dry weight equivalent of each soil sample was placed into a 25 ml beaker, which was placed into a vacuum desiccator with a new beaker containing 50 ml ethanol-free chloroform and a little zeolite. The same soil sample was placed as a blank into another desiccator without chloroform. In addition, a beaker containing 1M NaOH was placed into the desiccator to absorb the CO_2_ released from the soil during fumigation. The desiccator was sealed, and then the chloroform was boiled for 5 min by evacuating before incubating in the dark at 25 °C for 24 h. After fumigation, the beaker containing chloroform was removed, and the desiccator evacuated five times to remove the residual chloroform from the soil. The organic carbon of the fumigated and non-fumigated soils was extracted with 0.5M K_2_SO_4_. Finally, the carbon content of the K_2_SO_4_ extracts was measured using an Elementar total organic carbon (TOC) analyzer (Enviro TOC). The SMBC content (mg/kg) is calculated by multiplying the carbon increment caused by fumigation by a conversion factor (*K*_c_), which was 0.45, representing incomplete extraction of microbial carbon^30^.

### High-throughput sequencing and data processing

The metagenomic sequencing of the soil samples was performed using an Illumina NovaSeq PE150 platform at Wekemo Tech Co., Ltd., Shenzhen, China. To ensure data reliability, quality control and data processing of the raw sequencing data was performed: (1) Trimmatic software (v 0.39) was employed to remove 5′ and 3′ base sequences with a quality score lower than 20 (99% accuracy) and DNA sequences shorter than 50 bp and for keep high-quality pair and single-end reads^31^. (2) Bowtie2 software (v 2.3.5.1) was used to filter reads that were of host origin to get valid sequences (clean reads), which was further conducted quality testing using FastQC software for subsequent analysis^32,33^. (3) MEGAHIT software (v1.2.9) was used to assemble clean reads and got contigs after filtering host genes from soil samples (parameters: -min-contig-len 500)^34^. (4) The open reading frames (ORFs) of contigs were predicted using Prodigal software (v2.6.3), and all ORFs were clustered and constructed a non-redundant gene set using CD-HIT software (v4.8.1, de-redundant parameters: -c 0.95 -G 1)^33^. (5) Salmon software was applied to compare the non-redundant gene set with the clean reads to calculate the gene abundance of each sample^35^. (6) The non-redundant genes were translated into protein sequences using the transeq command of the Emboss software (v6.5.7) for subsequent alignment and annotation^36^.

After Illumina sequencing for 12 soil samples, approximately 128 GB of raw reads were generated. 386,458,752 clean reads and 3,129,907 non-redundant genes were generated, with an average of 32,204,896 reads and 260,825 non-redundant genes per sample by quality control and data processing. To evaluate the effect of aeolian deposition on soil carbon metabolism in the farmland, we studied the changes in microbial communities and carbon metabolism-related genes in soils with different amounts of sand deposition, including NR, KEGG metabolism pathway, and CAZy database^28^. DIAMOND software (v0.7.10.59) and BASTA software (v1.3.2.3) compared the non-redundant gene set with the three databases and obtained species annotation information and microbial carbon metabolism information of non-redundant genes^33^. In addition, we analyzed the differences in KEGG Orthology genes related to carbon metabolism in different sand deposition farmlands to evaluate the effects of sand deposition on these genes^37^. The CAZy database covers a family of enzymes that can catalyze carbohydrate degradation, modification, and biosynthesis, including Glycoside Hydrolases (GH), Glycoside Transferases (GT), Polysaccharide Lyases (PL), Carbohydrate Esterase (CE), Carbohydrate Binding Modules (CBM), and Auxiliary Activities (AA). Similarly, we also studied the effects of sand deposition on the CAZy genes.

### Data analyses and visual exhibition

The significant differences in the SMBC, CO_2_ emissions and physicochemical properties of soil samples were analyzed using a one-way analysis of variance (ANOVA) in SPSS 20.0. According to Tukey's test, a *P*-value < 0.05 was considered statistically significant. Principal Coordinate Analysis (PCoA) and analysis of similarity (ANOSIM) based on Bray–Curtis were applied to examine similarities and differences in microbial communities, KEGG metabolism pathways, and CAZy enzymes among the aeolian deposition soils. Canonical correspondence analysis (CCA) was used to classify the relationships between the soil microbial communities, KEGG metabolism pathways, CAZy enzymes, and the soil properties at a significance level of *P*-value < 0.05 using R software (v4.1.3). Mantel tests with 9999 permutations were performed using the R package vegan to evaluate the significant effects of the soil properties on the soil microbial communities, KEGG metabolism pathways, and CAZy enzymes. Pearson correlations analysis was further used to examine the relationships between the soil physicochemical properties and the microbial species, Carbohydrate metabolism, and CAZy enzymes.

To reveal the relationship between microbial communities and carbon metabolism functional characteristics, we analyzed and visualized the relative contributions of microbial communities to the KEGG metabolism pathways and CAZy enzymes in all soil samples. The Random Forest (RF) model determined the importance of each predicted variable using the rfPermute package in R software by assigning a random value to the predicted variable and calculating the increase in mean square error (%Inc MSE) between the observations and predictions^32^. This study used the RF model to identify carbon metabolism-related genes affected by sand deposition, including carbohydrate metabolism-related genes and CAZy genes. In addition, we analyzed the differences in the abundances of carbon metabolism-related genes under different amounts of sand deposition and the linear relationship between each gene and CO_2_ emissions.

## Results

### Changes in soil properties

Sand deposition significantly affected the soil particle size distribution in the farmland (*P* < 0.05) (Table S1). The sand content increased with the deposition amount, while clay and silt content decreased significantly. The average sand content in the DSD and MSD farmlands were 38.07% and 4.5% higher than in the CNSD farmland. In contrast, the clay and silt contents were 92.98% and 77.34% lower in the DSD farmland and 85.96% and 59.26% lower in the MSD farmland. Compared with the CNSD farmland, the average clay and silt content in the SSD farmland was 37.00% and 63.16% lower, respectively, while the sand content was 22.00% higher. The results indicated that sand deposition significantly reduced the content of soil fine particles and coarsened the soil texture of farmland.

The soil nutrient status gradually deteriorated with the increase in the sand deposition amount (Fig. 2). The soil nutrient contents at 0–15 cm depth in the DSD and MSD farmlands were significantly reduced (*P* < 0.05) compared with the control farmland. Specifically, in the DSD farmland, the content of TC reduced by 58.67%, SOC by 78.85%, DOC by 17.90%, TN by 62.13%, AN by 31.21%, TP by 42.06%, AP by 50.83%, and AK by 17.18%. Although the degree of reduction in the soil nutrients in the MSD farmland had decreased, the difference was still significant (*P* < 0.05). The soil nutrient contents in the SSD farmland were lower than in the CNSD, while no significant differences were observed except TC, SOC, and AP. Accumulated sand increased soil pH compared to the CNSD farmland, but only the DSD significantly differed (*P* < 0.05). The soil water content (SW) at 0–15 cm depth in the three sand deposition farmlands was significantly lower than in the control farmland (*P* < 0.05), and it decreased with the amount of sand deposition. Invertase, β-glucosidase, amylase, and cellulase activities in the 0–15 cm soil layer also showed significant changes. As the amount of sand deposition increased, the enzyme activities of the four enzymes significantly decreased. Compared with the CNSD farmland, the activities of invertase, β-glucosidase, amylase, and cellulase in the DSD farmland decreased by 66.64%, 64.97%, 29.58%, and 52.88%, respectively.

The nutrient contents in the CNSD and SSD farmlands significantly decreased with the increase in soil depth. In contrast, the nutrient contents in moderate and deep sand deposition farmlands decreased slightly or remained unchanged. Compared with the 0–15 cm depth, there were no significant differences in soil properties such as TC, SOC, AN, TP, TK, and pH in the 25–40 cm soil layer of DSD farmland (*P* > 0.05). The SW was significantly increased with the increase of soil depth and decreased with the increase of sand deposition amount. In addition, we found that as the depth of the soil layer increased, the effects of sand deposition on the soil properties gradually declined. Compared with the CNSD farmland, the TC, TN, SOC, AN, and AP contents in the 25–40 cm depth of the DSD farmland were decreased by 40.50%, 36.62%, 65.55%, 29.46%, and 69.37%, respectively. Nevertheless, significant differences in DOC, TP, AK, and pH were not observed among sand deposition farmlands. The enzyme activities of soil invertase, β-glucosidase, amylase, and cellulase significantly decreased with increasing soil depth (*P* < 0.05). However, the difference in the activities of carbon cycling enzymes among different sand deposition soils in the 25–40 cm soil layer decreased, and the activities of β-glucosidase and amylase were not significantly affected by sand deposition. In summary, the effects of sand deposition on the physicochemical properties of topsoil were higher than those of the deep soil. Therefore, we focused on studying the effects of sand deposition on the topsoil.

### Changes in soil CO_2_ emissions and microbial biomass carbon

**Figure 3:**
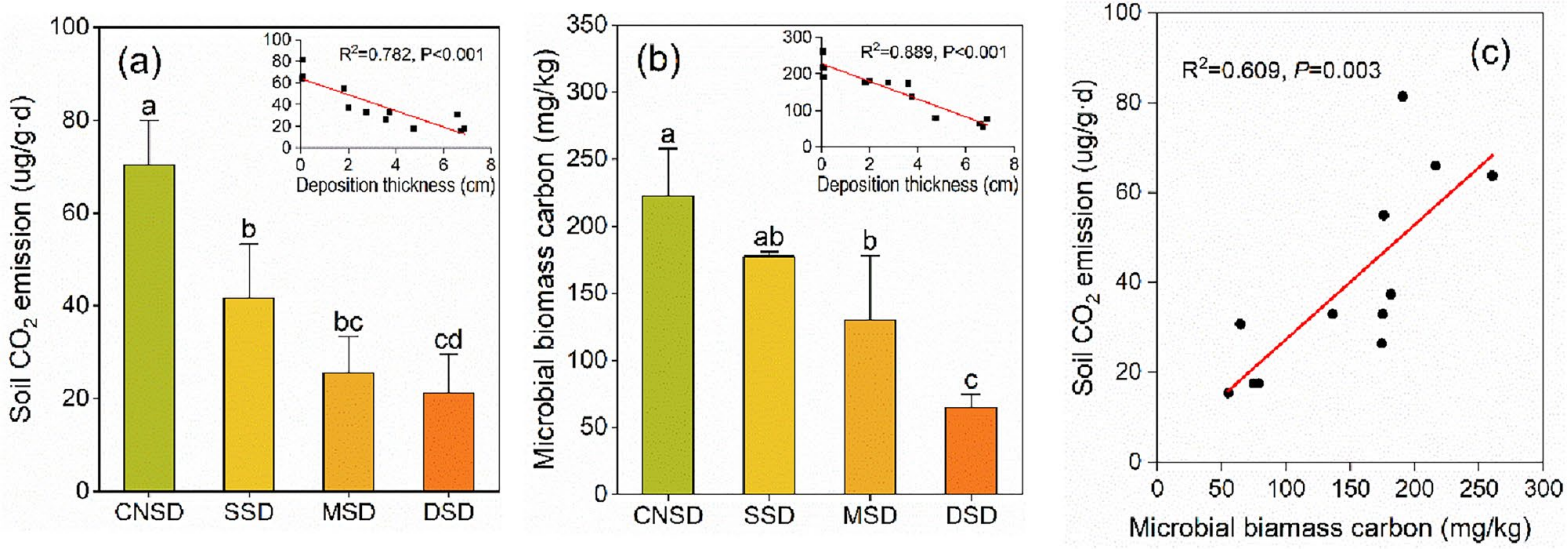
The soil CO_2_ emission (**a**) and microbial biomass carbon (**b**) in the aeolian deposition farmland and the linear relationship between soil CO_2_ emission and microbial biomass carbon (**c**). The significant differences (Tukey's test) at *P* < 0.05 were indicated by different letters.

Sand accumulation significantly affected the SMBC and carbon emissions in sand deposition farmland (*P* < 0.05) (Fig. 3). The SMBC and CO_2_ emissions significantly decreased with the increases in the amount of sand deposition. Compared with the CNSD farmland, the SMBC and CO_2_ emissions were reduced by 70.87% and 69.79% in the DSD farmland, respectively (Fig. 3a,b). The SMBC and CO_2_ emissions were negatively correlated with the amount of sand deposition (R^2^ = 0.782 and 0.889). We also found a linear positive correlation between soil CO_2_ emissions and SMBC (R^2^ = 0.609) (Fig. 3c).

### Changes in microbial community and carbon metabolism function

**Figure 4:**
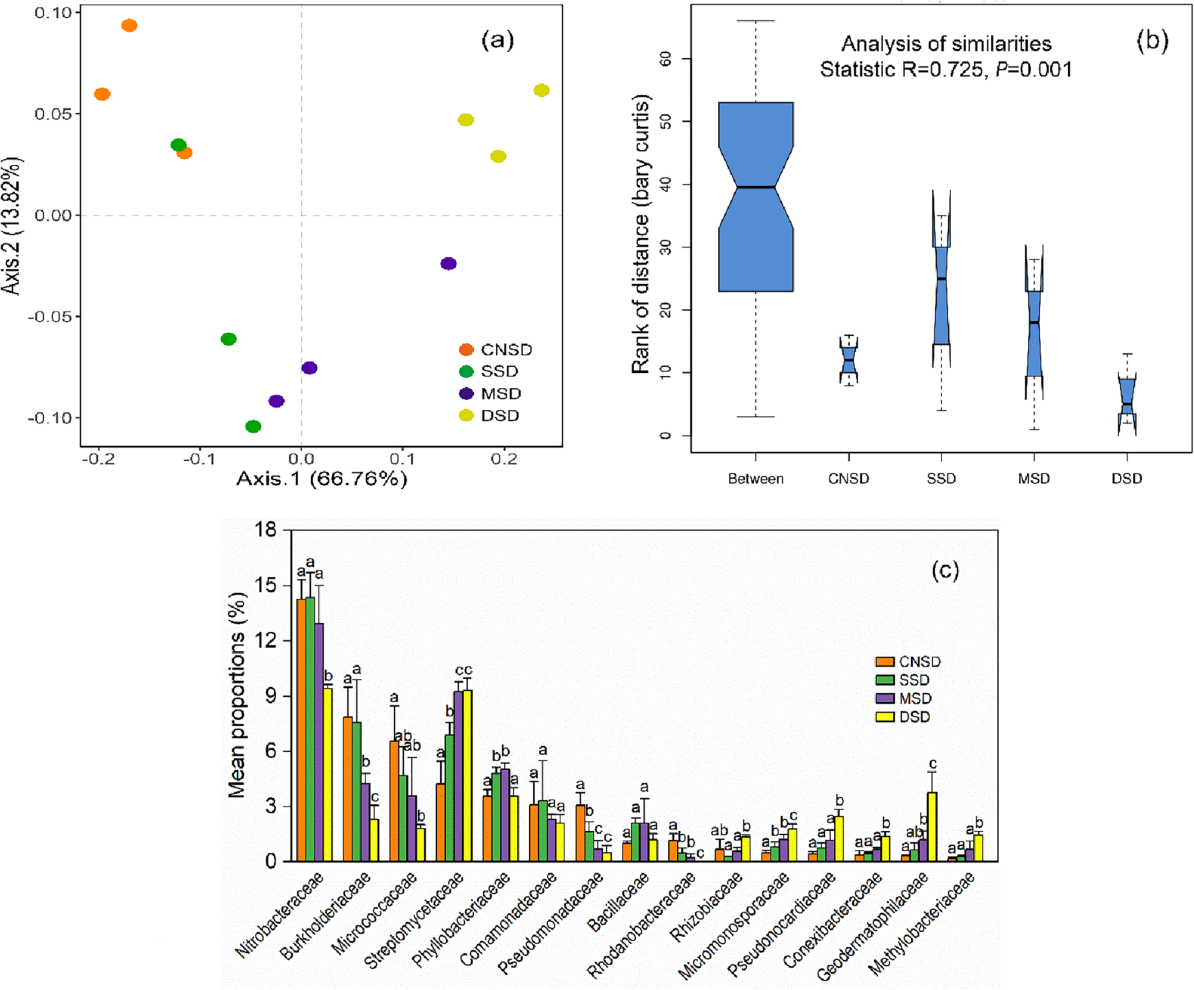
Principal coordinate analysis (PCoA) plot of the microbial communities at the family level in the aeolian deposition farmland (**a**). The significant differences among the microbial community structures were assessed through the analysis of similarities (ANOSIM) (**b**), and Tukey's test analyzed the significant differences in the relative abundance of each taxon (**c**), indicated as different letters (*P* < 0.05).

The result of PCoA indicated the significant composition change of the soil microbial community along the sand deposition gradient from the CNSD to DSD farmland (Fig. 4a). The ANOSIM test showed that the soil microbial community in the SSD, MSD, and DSD farmlands significantly different from in the control farmland (Fig. 4b; R = 0.725, *P* = 0.001). Sand deposition significantly increased the relative abundances of Streptomycetaceae, Geodermatophilaceae, and Methylobacteriaceae. In contrast, the relative abundances of Nitrobacteraceae, Burkholderiaceae, and Rhodanobacteraceae in the deep deposition farmland were significantly lower than in the control farmland (Fig. 4c). The Shannon index was used to evaluate the α-diversity of microbial communities (Fig. S1). The Shannon indexes of bacteria and fungi in the DSD farmland were significantly lower than in the other farmlands (*P* < 0.05), indicating that deep sand deposition significantly impacted soil microbial community diversity.

We also studied the differences in KEGG metabolism pathways and CAZy enzymes of soil microorganisms in the sand deposition farmland. The results indicated the significant functional changes of the microbial metabolism along the sand deposition gradient from the CNSD to DSD farmland, including the metabolism pathway at KEGG level 1 with the ANOSIM result of R = 0.704, *P* = 0.001 (Fig. S2a,b) and the class level of CAZy with the ANOSIM result of R = 0.688, *P* = 0.002 (Fig. S3a,b). Compared to the CNSD farmland, the carbohydrate and amino acid metabolisms in KEGG metabolism pathways were decreased by 8.91% and 2.57% in the DSD farmland (Fig. S2c). Still, carbohydrate metabolism was the highest metabolism pathway in the farmlands. Therefore, we focused on studying carbon metabolism in the aeolian deposition farmland. At the class level of CAZy, sand deposition significantly decreased the gene abundances of GH and CBM but increased those of GT and CE (Fig. S3c). Compared to the CNSD farmland, the changes of GH, CBM, GT and CE were -7.32%, -24.32%, 9.39% and 52.50% in the DSD farmland. Nevertheless, the changes in the gene abundances of PL and AA were insignificant (*P* > 0.05).

### Correlations between microbial community, carbon metabolism function, and soil properties

**Figure 5:**
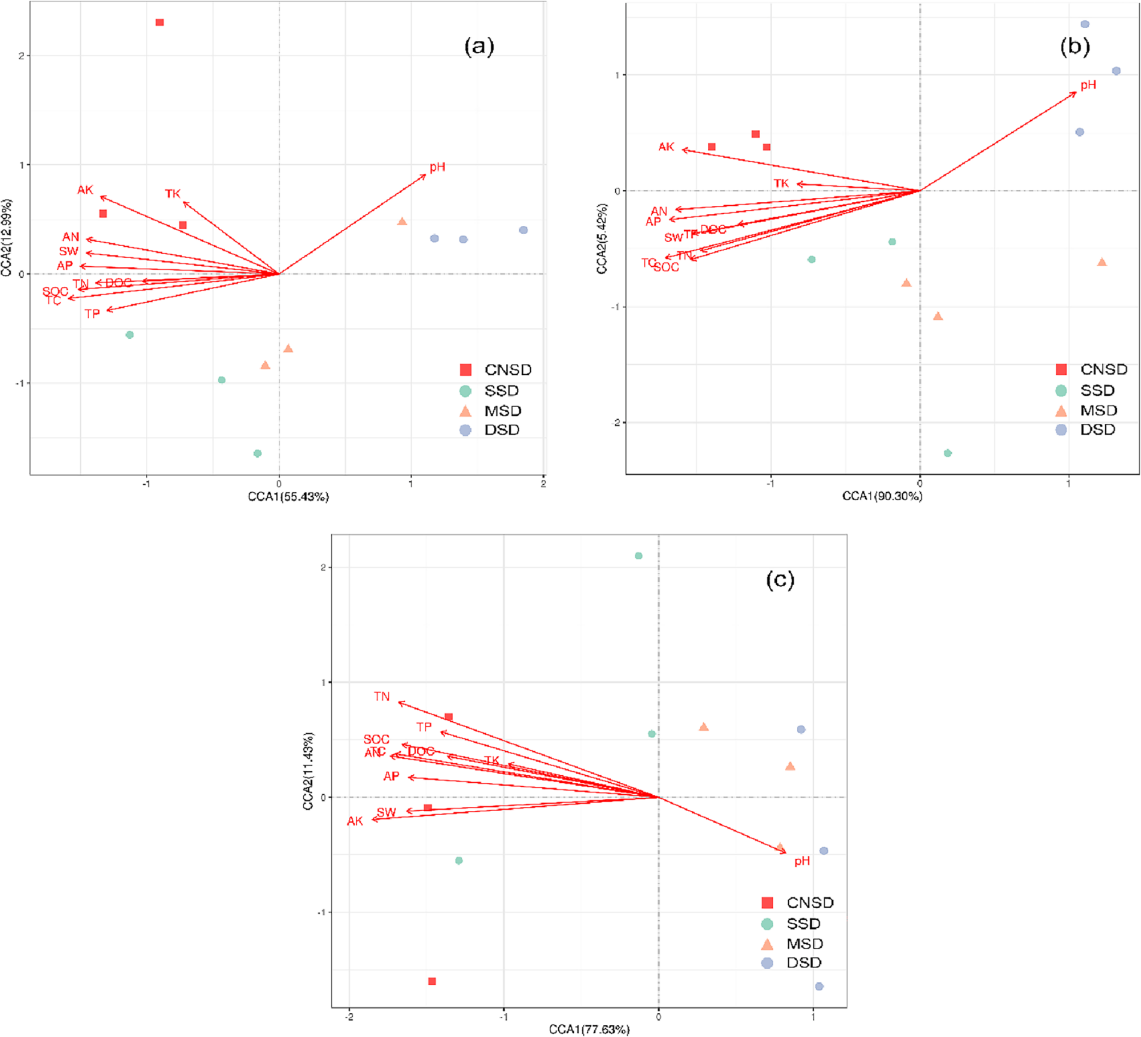
The correlations between the soil physicochemical properties and microbial communities (**a**), metabolism pathway at KEGG level 1 (**b**), and CAZy enzymes (**c**) were analyzed by Canonical Correspondence Analysis (CCA).

CCA and Mantel tests identified the correlations between soil properties, microbial community, and carbon metabolism function. The combined variables of the first two axes of CCA explained 68.42% of the variance of the microbial community (Fig. 5a), 95.72% of the variance of the metabolism pathway at KEGG level 1 (Fig. 5b), and 89.06% of the variance of CAZy at the class level (Fig. 5c) in the sand deposition farmland, respectively.

Mantel tests indicated that soil TC, TN, SOC, DOC, AN, TP, AP, AK, pH, and SW were significantly associated with the class level of CAZy, with R^2^ ranging from 0.26 (AK) to 0.68 (SOC) (Table S2). Similarly, soil TC, TN, SOC, AN, TP, AP, AK, pH, and SW were significantly associated with microbial communities with R^2^ ranging from 0.32 (pH) to 0.74 (TC). TC, TN, SOC, DOC, AN, TP, AP, AK, and SW were significantly associated with the metabolism pathway at KEGG level 1, with R^2^ ranging from 0.44 (AP) to 0.70 (SOC).

Furthermore, the relative abundances of Burkholderiaceae, Micorcoccaceae, Pseudomonadaceae, and Rhodanobacteraceae were significantly positively correlated with soil nutrient contents containing TC, TN, SOC, DOC, AN, TP, and AP (*P* < 0.05) (Table S3). In contrast, the relative abundances of Streptomycetaceae, Micromonosporaeae, Pseudonocardiaceae, Geodermatophilaceae, Conexibacteraceae, and Methylobacteriaceae were negatively correlated with them (*P* < 0.05). Microbial carbon metabolism functional genes containing carbohydrates, GH, CE, and CBM showed positive correlations with soil nutrient contents (Table S3) (*P* < 0.05). The decreased soil carbon content in the sand deposition farmland reduced the carbon metabolism-related genes, including carbohydrate metabolism and carbohydrate-active enzymes.

### Correlations between microbial community and carbon metabolism function

**Figure 6:**
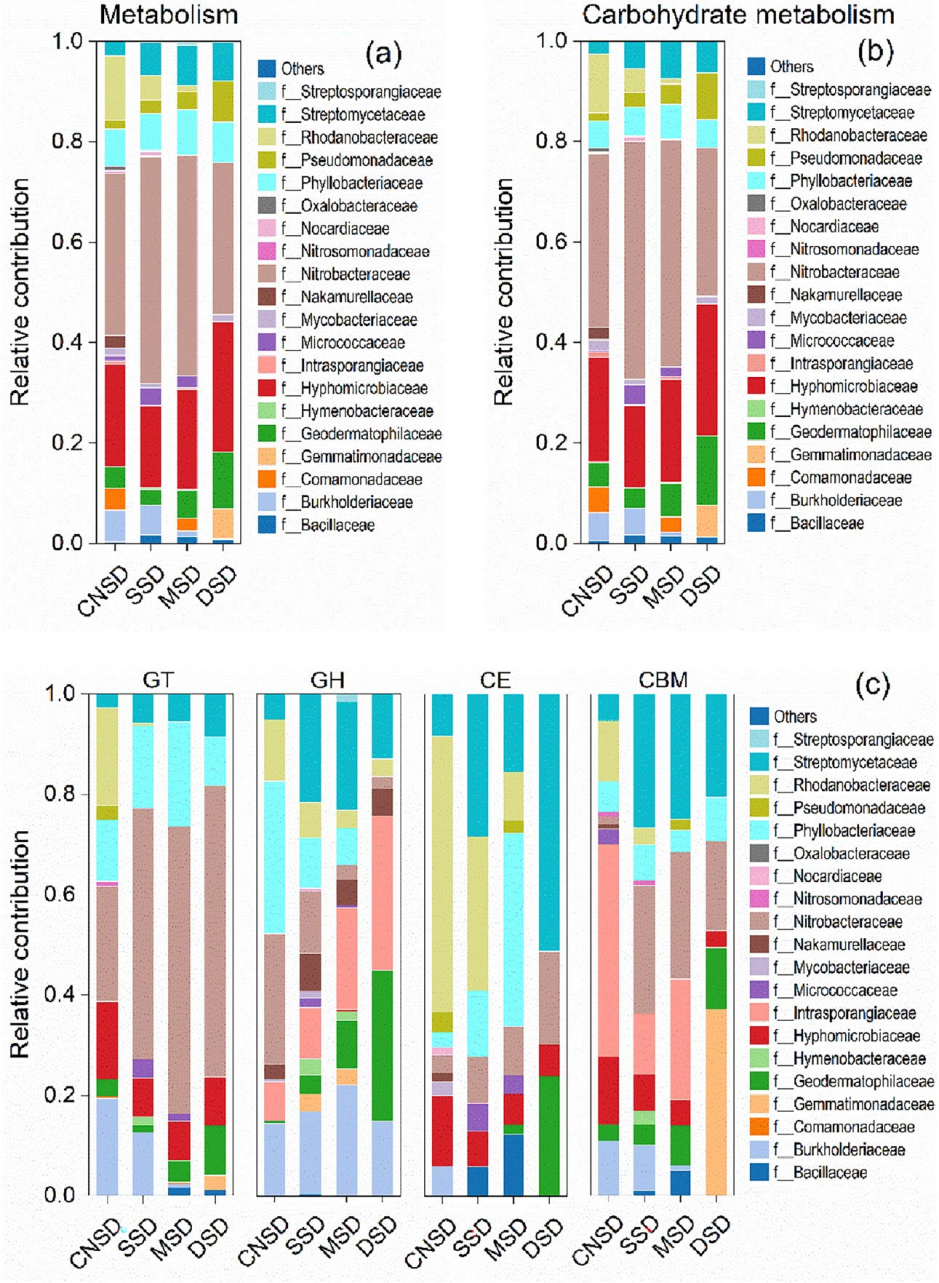
The relative contributions of microbial communities at the family level to the metabolism pathway (**a**), carbohydrate metabolism (**b**), and CAZy enzymes at the class level (**c**) in the aeolian deposition farmland.

Figure 6 showed that the relative contributions of Streptomycetaceae, Pseudonocardiaceae, and Geodermatophyllaceae to KEGG metabolism and carbohydrate metabolism increased with the sand deposition amount. In contrast, the relative contributions of Burkholderiaceae, Nitrobacteraceae, and Rhodanobacteraceae decreased. Compared to the CNSD farmland, the relative contributions of Streptomycetaceae, Pseudonocardiaceae, and Geodermatophilaceae in the DSD farmland to the KEGG metabolism increased by 4.82%, 6.40%, and 6.96% (Fig. 6a) and to the carbohydrate metabolism increased by 3.87%, 7.62% and 8.81% respectively (Fig. 6b). In contrast, the relative contributions of Burkholderiaceae, Nitrobacteraceae and Rhodanobacteraceae in the DSD farmland to the KEGG metabolism decreased by 6.02%, 1.96%, and 12.66%, and to the carbohydrate metabolism decreased by 5.49%, 5.07% and 11.77%, respectively.

Similarly, the relative contributions of the four bacteria, Streptomyceaceae, Geodermatophilaceae, Burkholderiaceae, and Rhodanobacteraceae, to the carbohydrate-active enzymes also exhibited regular changes with the increased deposition amount (Fig. 6c). Compared to the CNSD farmland, the relative contributions of Streptomycetaceae and Geodermatophilaceae in the DSD farmland to the carbohydrate-active enzymes increased by 5.89% and 6.55% (GT), 7.70%, and 29.67% (GH), 42.87% and 23.85% (CE), and 15.31% and 9.15% (CBM), but Burkholderiaceae and Rhodanobacteraceae reduced by 19.42% and 19.46% (GT), 0.33% and 8.53% (GH), 5.92% and 54.93% (CE), and 11.01% and 12.14% (CBM). The results indicated that Streptomycetaceae, Geodermatophyllaceae, Burkholderiaceae, and Rhodanobacteriaceae played a crucial role in soil C metabolism in sand deposition farmland.

### Carbon metabolism-related genes regulate carbon emissions

**Figure 7:**
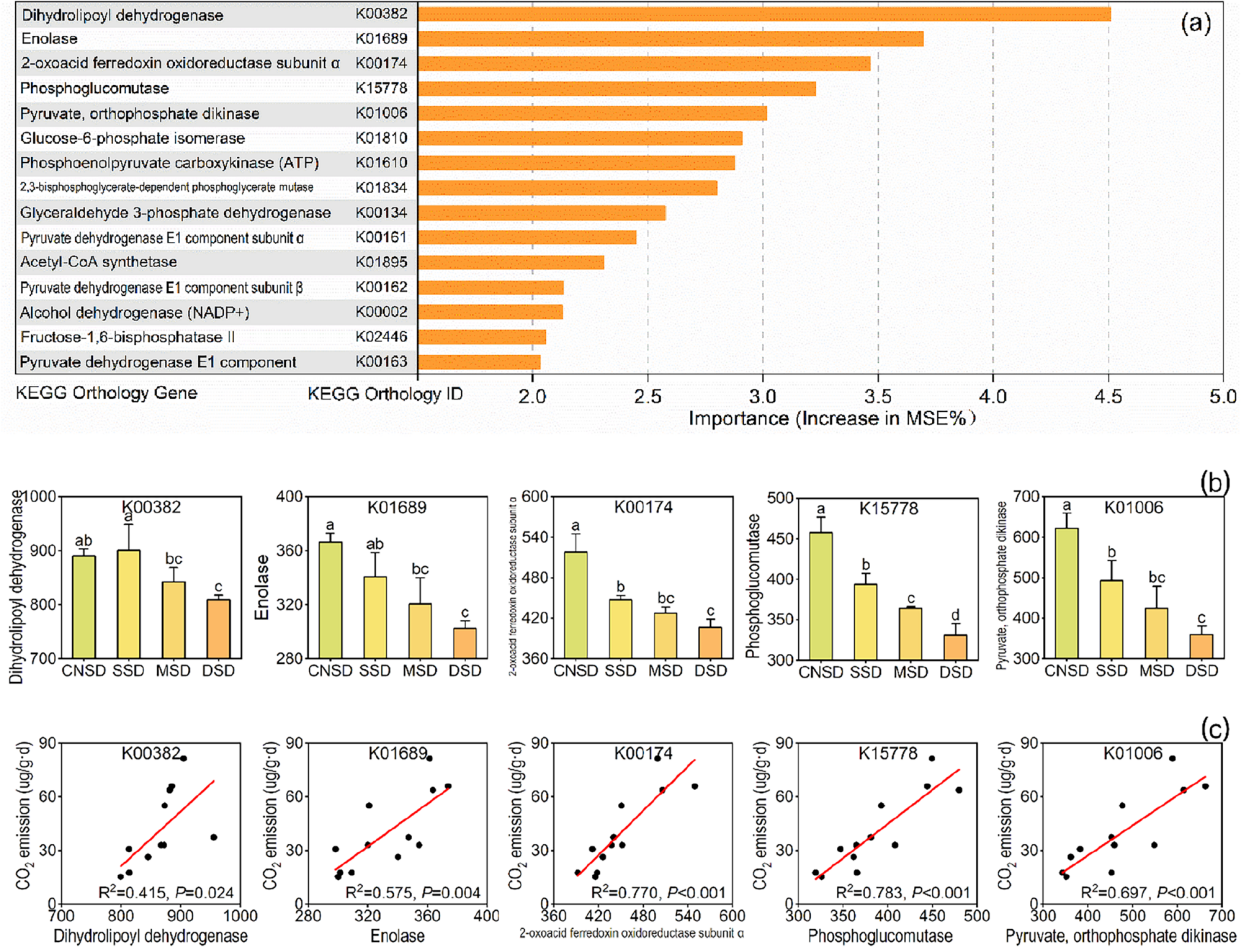
The significant genes of carbohydrate metabolism were analyzed by Random Forest (*P* < 0.05) (**a**). Subgraphs include the abundance differences of selected functional genes in the aeolian deposition farmland (**b**) and the linear relationships between CO_2_ emission and selected functional genes (**c**). *P* < 0.05 indicated a significant correlation.

**Figure 8:**
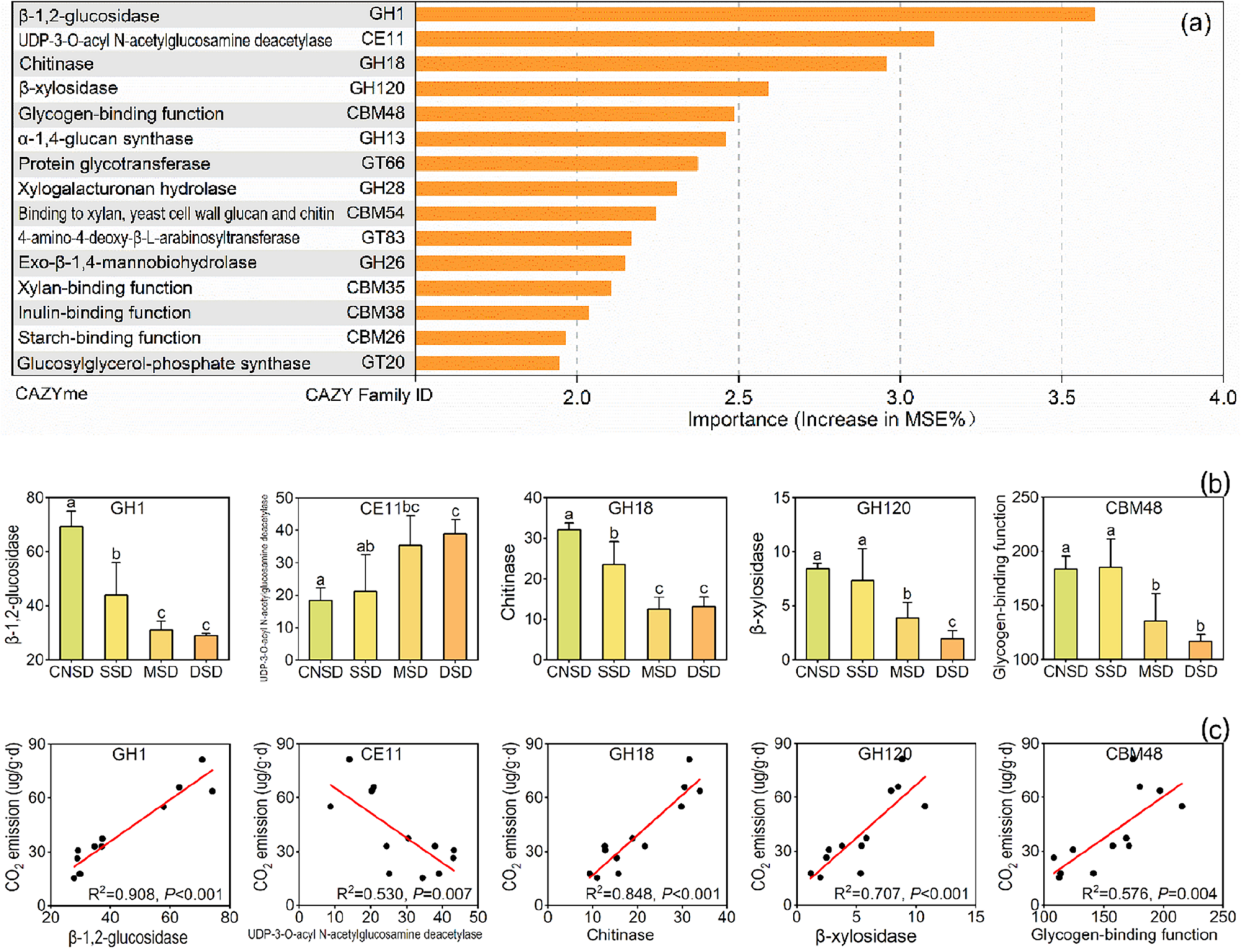
The significant genes of carbohydrate-active enzymes were analyzed by Random Forest (*P* < 0.05) (**a**). Subgraphs include the abundance differences of selected functional genes in the aeolian deposition farmland (**b**) and the linear relationships between CO_2_ emission and selected functional genes (**c**). *P* < 0.05 indicated a significant correlation.

The RF model was used to identify the most critical carbohydrate metabolism-related enzymes (Fig. 7a) and carbohydrate-active enzymes (Fig. 8a) that significantly affect CO_2_ emissions in the sand deposition farmland. The results indicated that the abundances of the five most crucial carbohydrate metabolism-related enzyme genes, including the genes encoding dihydrolipoyl dehydrogenase (K00382), enolase (K01689), 2-oxoacid ferredoxin oxidoreductase subunit α (K00174), phosphoglucomutase (K15778), and pyruvate-orthophosphate dikinase (K01006), significantly decreased with the increased deposition amount and the reduced soil CO_2_ emission (Fig. 7b and c). The most critical fifteen carbohydrate-active enzyme genes at the CAZy-family level were all from the CH, GT, CE, and CBM class (Fig. 8a). The abundances of genes encoding β-1,2-glucosidase (GH1), Chitinase (CH18), β-xylosidase (CH120) and Glycogen-binding modules (CBM48) decreased along with the increased sand deposition amount and the reduced soil CO_2_ emission (Fig. 8b and c). In contrast, the abundance of gene encoding UDP-3-O-acyl *N*-acetylglucosamine deacetylase (CE11) significantly increased in the sand deposition farmland.

## Discussion

The quality and health status of soil affects crop growth, water source protection, and ecosystem stability^38^. In this study, the sand content in the DSD and MSD farmlands was significantly higher, and the clay and silt contents were lower than in the CNSD farmland, thus increasing the soil roughness^39^. Our research shows that sand mainly accumulated on the windward side and was an essential reason for the coarsening of soil texture in farmland, which was consistent with the results of Zhao et al.^26^. The correlation analysis showed a significant negative correlation between soil sand and SW (R^2^ = 0.815). Compared with the CNSD farmland, the average SW of the 0–15 cm layer in the DSD and MSD farmlands decreased by 50.72–6.06% and 26.18–9.07%, indicating soil roughness was related to soil water storage capacity. In addition, the changes in soil nutrients were affected by sand accumulation and significantly decreased with the increase of aeolian deposition during wind erosion. The soil nutrients, including TC, SOC, DOC, TN, AN, TP, AP, and AK, were negatively correlated with soil sand (Table S4).

Previous studies showed that the soil nutrient in wind erosion farmland significantly decreased^13^, and the lost SOC would accumulate in sand accumulation areas^21^. However, this study suggested that SOC did not increase with sand deposition. Compared with the CNSD farmland, the average SOC content in the 0–15 cm layer in the DSD and MSD farmlands decreased by 78.85% and 41.39%, respectively. The reduced SOC in sand deposition may be due to several reasons. Soil aggregates were broken by wind erosion and released SOC and microorganisms^40^, increasing microbial metabolic activity and SOC mineralization rate. SOC in soil particles was degraded into small molecule compounds through photocatalysis and natural oxidation during the soil particles migration with wind, which may result in SOC loss of over 20%^41^. In addition, the fewer fine particles in the aeolian deposition were also an essential reason for SOC loss, as SOC usually combined with them^42,43^.

The carbon emissions in sand deposition farmland were significantly decreased, unlike wind erosion farmland^12^. Both the loss of soil nutrients and the decrease in microbial biomass caused by the deterioration of soil texture were essential reasons for the decrease in carbon emissions^44,45^. Soil microorganisms, which regulate soil nutrient cycling and plant productivity, are important indicators of soil health^46^. Soil organic matter, SW, and microorganisms were the main limiting factors for plant productivity in agricultural ecosystems^47^. Soil degradation caused by sand accumulation inevitably slows crop growth, reduces crop yield, and seriously affects soil ecosystems and agricultural productivity^11^.

Both the classification and functional structure of microbial communities were influenced by sand deposition, indicating their sensitivity to environmental changes^21^. Significant changes occurred in the microbial community as the topsoil was migrated from wind erosion plots to sand deposition plots. This study found that although the relative abundance of dominant microorganisms in the DSD farmland significantly decreased, such as Nitrobacteraceae, Burkholderiaceae, and Rhodanobacteraceae, the relative abundance of two actinomycetes, Streptomycetaceae and Geodermatophilaceae, increased by 101.04% and 975.21%, respectively, indicating that soil microorganisms adapted to adverse environments through self-regulation of species abundance during the wind erosion and sand accumulation process. Some studies also confirmed that sandy soils with rampant soil erosion were rich in actinomycetes but contained fewer γ-proteobacteria than other microbial communities^48^. Microorganisms spread over long distances through dust particles and aerosols, causing significant changes in the air and soil microorganisms in downwind areas^10^. Therefore, the increased actinomycetes in the sand deposition farmlands in this study may originate from the wind erosion and migration of sand dust particles in the upwind zone. In addition, changes in microbial community structure may depend on other environmental factors. The Actinobacteria phylum contains many decomposing microbes and plays an essential role in the degradation process of animal and plant residues^49^. In this study, farmlands often used straw returning and stubble retention to resist wind erosion and soil degradation, which provided sufficient nutrients for actinomycetes and may be an essential reason for their survival. Meanwhile, the hyphae and spores produced by most actinomycetes can adapt to environmental stresses such as drought and nutrient deficiency^50^.

The unique physiological characteristics of actinomycetes ensure that they can survive in arid and barren soil and play a role in soil carbon cycling^51,52^. Several studies showed that actinomycetes resisted erosion through various biophysical protection methods. Extracellular polysaccharides (EPs) secreted by actinomycetes bound soil particles together^53^ and formed polysaccharide-polycation bridges, improving soil tensile strength and wear resistance and alleviating the destructive nature of erosion^54^. Filamentous bacteria can stabilize soil structure by crosslinking and entangling soil particles with mycelium^55,56^. Yang et al.^57^ confirmed that the abundance of actinomycetes in farmland was positively correlated with micro-aggregates stability (0.25–0.053 mm) and erosion resistance. In addition, Actinobacteria and their spores have stronger stress resistance, such as salt alkali, drought, and high-temperature resistance^58^. The diverse metabolic types of actinomycetes provide their ability to increase soil fertility and reduce the harm caused by environmental changes through mineral dissolution and organic matter decomposition, mineralization, and storage^52,59^. The unique physiological and metabolic characteristics of Actinobacteria were the reasons for the higher relative abundance of Actinobacteria in the sand deposition farmland than that in the non-sand deposition farmland in this study.

Soil microbial biomass carbon is one indicator for evaluating the quantity and activity of soil microorganisms^60^. Although the SMBC accounts for a small proportion of the soil carbon pool, it directly participates in soil biochemical processes. It is closely related to soil nutrient cycling, such as C, N, P, and S^61^. In this study, sand deposition significantly decreased the SMBC in farmland (Fig. 3b). The accumulated sand inhibited the reproduction and metabolism of soil microorganisms, reduced the content of active organic carbon in the soil, and harmed the stability of SOC^62^. Since the increased soil carbon sequestration was mainly driven by SMBC^63^, the soil carbon pool was not increased due to the continuously accumulated sand but decreased. The decrease in the quantity and activity of soil microorganisms caused by sand deposition may be essential reasons for soil degradation^64^.

Research showed that wind erosion significantly reduced soil microbial biomass and activity, thereby decreasing the abundance of soil carbon metabolism genes and inhibiting soil carbon cycling^21^. However, to our knowledge, functional genes related to carbon metabolism in aeolian deposition farmland have been rarely analyzed. This study found that the relative contribution of actinomycetes to soil carbohydrate metabolism and carbohydrate active enzymes significantly increased in aeolian deposition farmland, suggesting that actinomycetes provided positive feedback to adverse environments by increasing carbon metabolism levels^59,65^. Nevertheless, the overall carbohydrate metabolic activity in the aeolian deposition farmland was still relatively lower. Compared with the CNSD farmland, the gene abundance of carbohydrate metabolism in the DSD and MSD farmlands decreased by an average of 8.91% and 5.62%. Correlation analysis showed that carbohydrate metabolism was significantly influenced by soil nutrient content (Table S3). Enzymes related to carbohydrate metabolism in the sand deposition farmland, such as enolase, phosphoglucose mutase, and pyruvate phosphokinase, significantly decreased in gene abundance with increasing deposition amount. These enzymes were mainly involved in glycolysis and energy metabolism^66^, indicating that sand accumulation hindered the microbial energy-yielding metabolism. Research showed that microorganisms migrating in the air may have specific metabolic characteristics of resistance to dryness, such as resistance to environmental stressors and the production and degradation of polysaccharides^9,67^. Therefore, microorganisms in the sand deposition farmlands may possess these special metabolic activities. This study found that the abundance of CE11 significantly increased in the DSD farmland. UDP-3-O-acyl-N-acetylglucosamine deacetylases (LpxC, EC 3.5.1.-) are essential members of the CE11 family, which is a crucial enzyme involved in the synthesis of lipid A as well as the main component of lipopolysaccharides in the outer membrane of Gram-negative bacteria^67^. Bacteria enhanced the synthesis of cell wall lipopolysaccharides by increasing the abundance of LpxC. The significant increase in the abundance of gram-negative bacteria, such as rhizobiaceae and methylobacteriaceae, may be related to improving cell wall metabolic pathways. LpxC may be a marker metabolite in sand deposition soil, and further research was needed.

Carbohydrate active enzymes (CAZymes) are essential in complex carbohydrate metabolism and soil carbon turnover processes^68,69^. In the sand deposition farmland, the abundance of genes encoding carbohydrate-active enzymes related to hydrolyze cellulose, xylan, and chitin, such as β-1,2-glucosidase (GH1), chitinase (GH18) β-Xylosidase (GH120), and Xylan Binding Module (CBM54), decreased with the increase of sand deposition amount. Several studies showed that the glycoside hydrolases (GH) family was crucial for the degradation of lignocellulose^68,70^ and played essential roles in soil humification^71^. The decrease in GH enzyme abundance in the sand deposition farmland would inevitably reduce the soil humification index^70^, which was not conducive to the carbon conversion of straw, plant residues, and microbial residues^69^. These gene predictive factors were related to soil carbon metabolism function and may be important biomarkers for aeolian deposition farmland, requiring further research on large sample sizes or spatial scales. Sand deposition altered the abundance of genes related to carbon metabolism in sensitive microorganisms and their relative contribution to carbon metabolism. These sensitive carbon metabolism genes and active enzymes perhaps were potential ecological indicators for soil in aeolian deposition farmland. The consistency of these observations results in other areas of wind erosion-aeolian deposition farmland in China needs further research.

## Conclusions

This work is one of the earliest studies on the changes of soil microorganisms and carbon metabolism functional genes in aeolian deposition farmland. Long-term sand deposition significantly changed the soil properties of farmland, including a decrease in soil nutrients, SOC, clay content, and water content, as well as an increase in sand content and pH value, leading to soil infertile, roughness, and dryness, and ultimately causing the deterioration of land resources. Severe sand deposition resulted in a serious decline in soil nutrients and water storage capacity and altered soil microbial community structure and carbon metabolism functions, such as a decrease in α-Proteobacteria and an increase in Actinobacteria. It also decreased the soil microbial diversity, carbohydrate metabolism, and carbohydrate-active enzyme. These changes decreased soil carbon emissions, which may affect soil carbon cycling in farmland and lead to more severe land degradation. The changes in soil properties and carbon metabolism function caused by sand deposition perhaps profoundly affect agricultural ecosystems and global soil carbon balance.

### Supplementary Information

Supplementary Information.

## Data Availability

The datasets presented in this study can be found on an online public repository: https://www.ncbi.nlm.nih.gov/bioproject/PRJNA1083081.
