# Supplementary material for: Effects of aeolian deposition on soil properties and microbial carbon metabolism function in farmland of Songnen Plain, China
Source: Sci Rep. 2024 Jun 26;14:14791. doi: 10.1038/s41598-024-65578-0 (PMC11208439; doi:10.1038/s41598-024-65578-0)
Supplement: Supplementary file 1 — Supplementary Information. [file 41598_2024_65578_MOESM1_ESM.docx]

**Supplementary information**

**Effects of aeolian deposition on soil properties and microbial carbon metabolism function in farmland of Songnen Plain, China**

Jixian Mo ^1, 2^, Ziwei Song ^2^, Yanjing Che ^1^, Jie Li ^2^, Tianyi Liu ^2^, Jingyi Feng ^1^, Ziying Wang ^1^, Jiandong Rong ^3^, and Siyu Gu ^1,^ *

^1^ College of Resources and Environment, Northeast Agricultural University, Harbin 150030, China

^2^ College of Life Science and Agriculture and Forestry, Qiqihar University, Qiqihar 161006, China

^3^ Qiqihar Experimental Station, Heilongjiang Province Hydraulic Research Institute, Qiqihar 161006, China

*Corresponding author

E-mail: [gusiyu@neau.edu.cn](mailto:gusiyu@neau.edu.cn) (Siyu Gu)

Table S1. The amount of sand deposition and soil texture at sampling quadrats and the significant differences (Tukey’s test) at *P*<0.05 were indicated by different letters.

| Sample belt | 1 | | | 2 | | | 3 | | | 4 | | |
| --- | --- | --- | --- | --- | --- | --- | --- | --- | --- | --- | --- | --- |
| Sampling quadrat | 1 | 2 | 3 | 4 | 5 | 6 | 7 | 8 | 9 | 10 | 11 | 12 |
| Average deposition thickness (cm) | 2.60±0.22 | 2.42±0.16 | 2.48±0.13 | 1.55±0.13 | 1.30±0.13 | 1.13±0.21 | 0.85±0.10 | 0.62±0.13 | 0.67±0.06 | 0.00±0.05 | 0.02±0.08 | 0.03±0.03 |
| Soil bulk density (g/cm^3^) | 1.30±0.05 | 1.29±0.03 | 1.34±0.01 | 1.26±0.02 | 1.24±0.04 | 1.24±0.05 | 1.15±0.04 | 1.14±0.05 | 1.11±0.06 | 1.05±0.04 | 1.08±0.03 | 1.07±0.03 |
| Sand deposition amount (kg/m^2^) | 33.60±1.69 | 31.07±2.16 | 33.30±2.72 | 19.45±1.44 | 16.08±1.40 | 13.99±2.10 | 9.79±1.02 | 7.01±1.15 | 7.35±0.25 | 0.01±0.52 | 0.19±0.84 | 0.35±0.31 |
| Sand deposition rate (kg/m^2^‧d) | 0.56±0.03 | 0.52±0.04 | 0.56±0.05 | 0.32±0.02 | 0.27±0.02 | 0.23±0.03 | 0.16±0.02 | 0.12±0.02 | 0.12±0.00 | 0.00±0.01 | 0.00±0.01 | 0.01±0.01 |
| Sand Deposition class | Deep (DSD) | | | Moderate (MSD) | | | Shallow (SSD) | | | None (CNSD) | | |
| Sand (0.02-2.00 mm) (%) | 92.63±0.25d | | | 86.74±0.81c | | | 81.85±2.77b | | | 67.09±0.80a | | |
| Silt (0.002-0.02 mm) (%) | 7.33±0.24d | | | 13.18±0.87c | | | 20.38±1.85b | | | 32.35±0.76a | | |
| Clay (≤0.002 mm) (%) | 0.04±0.02c | | | 0.08±0.08c | | | 0.21±0.17b | | | 0.57±0.04a | | |

Table S2. The significant effects of soil physicochemical properties on the microbial communities, the KEGG level 1 of metabolic pathway, and the class level of CAZy were analyzed using the Mantel Test with 9999 permutations. *P* <0.05 indicated the significance effect.

| Variables | Soil microbial communities | | KEGG metabolic pathways | | CAZy enzymes | |
| --- | --- | --- | --- | --- | --- | --- |
|  | R^2^ | *P* | R^2^ | *P* | R^2^ | *P* |
| TC | 0.74 | 0.001 | 0.58 | 0.005 | 0.47 | 0.007 |
| SOC | 0.63 | 0.001 | 0.70 | 0.001 | 0.68 | 0.001 |
| DOC | *0.12* | *0.244* | 0.63 | 0.001 | 0.44 | 0.007 |
| TN | 0.49 | 0.001 | 0.56 | 0.002 | 0.41 | 0.016 |
| AN | 0.53 | 0.002 | 0.62 | 0.001 | 0.58 | 0.001 |
| TP | 0.34 | 0.037 | 0.48 | 0.001 | 0.45 | 0.004 |
| AP | 0.64 | 0.001 | 0.44 | 0.007 | 0.51 | 0.003 |
| TK | *-0.12* | *0.679* | *-0.14* | *0.806* | *-0.20* | *0.940* |
| AK | 0.72 | 0.002 | 0.47 | 0.005 | 0.26 | 0.045 |
| pH | 0.32 | 0.044 | *0.17* | *0.101* | 0.27 | 0.038 |
| SW | 0.44 | 0.009 | 0.48 | 0.004 | 0.47 | 0.002 |

Table S3. Pearson correlation between the soil physicochemical properties and the microbial species, Carbohydrate metabolism, and CAZy enzymes (GH, CE, GT, and CBM). *Correlation is significant at the 0.05 level, **significant at the 0.01 level, and ***significant at the 0.001 level.

| Items | TC | SOC | DOC | TN | AN | TP | AP | TK | AK | pH | SW |
| --- | --- | --- | --- | --- | --- | --- | --- | --- | --- | --- | --- |
| Nitrobacteraceae | 0.671* | 0.622* | *0.476* | 0.637* | *0.49* | *0.538* | *0.566* | *0.357* | *0.35* | -0.711** | *0.573* |
| Burkholderiaceae | 0.839*** | 0.818** | 0.692* | 0.774** | 0.846*** | 0.699* | 0.818** | 0.448 | 0.769** | *-0.56* | 0.895*** |
| Micrococcaceae | 0.86*** | 0.762** | 0.692* | 0.76** | 0.657* | 0.86*** | 0.713** | *0.245* | *0.517* | -0.743** | 0.664* |
| Streptomycetaceae | -0.881*** | -0.797** | -0.783** | -0.711** | -0.741** | -0.762** | -0.748** | *-0.322* | -0.727** | *0.441* | -0.825*** |
| Pseudomonadaceae | 0.972*** | 0.916*** | 0.79** | 0.816** | 0.783** | 0.86*** | 0.797** | *0.175* | 0.671* | -0.63* | 0.881*** |
| Rhodanobacteraceae | 0.902*** | 0.825*** | 0.741** | 0.774** | 0.867*** | 0.853*** | 0.944*** | *0.301* | 0.692* | -0.63* | 0.888*** |
| Micromonosporaceae | -0.888*** | -0.846*** | -0.657* | -0.788** | -0.832*** | -0.755** | -0.874*** | *-0.273* | -0.72** | 0.564 | -0.902*** |
| Pseudonocardiaceae | -0.895*** | -0.818** | -0.713** | -0.774** | -0.86*** | -0.846*** | -0.93*** | *-0.329* | -0.699* | 0.623* | -0.881*** |
| Conexibacteraceae | -0.853*** | -0.769** | -0.769** | -0.858*** | -0.692* | -0.867*** | -0.727** | *-0.503* | -0.58* | 0.701* | -0.741** |
| Geodermatophilaceae | -0.853*** | -0.797** | -0.678* | -0.788** | -0.846*** | -0.713** | -0.888*** | *-0.406* | -0.727** | *0.574* | -0.895*** |
| Methylobacteriaceae | -0.832*** | -0.727** | -0.615* | -0.764** | -0.755** | -0.692* | -0.902*** | *-0.329* | -0.594* | 0.616* | -0.804** |
| Carbohydrate metabolism | 0.881*** | 0.867*** | 0.881*** | 0.799** | 0.895*** | 0.815** | 0.79** | *0.144* | 0.839*** | *-0.434* | 0.874*** |
| CBM | 0.706* | 0.741** | 0.776** | 0.613* | 0.727** | 0.631* | *0.517* | *0.06* | 0.727** | *-0.235* | 0.734** |
| CE | -0.685* | -0.79** | -0.65* | -0.715** | -0.685* | -0.582* | *-0.371* | *0.053* | -0.727** | *0.357* | -0.657* |
| GH | 0.713** | 0.762** | 0.818** | 0.595* | 0.776** | 0.628* | *0.517* | *-0.053* | 0.79** | *-0.151* | 0.762** |
| GT | *0.364* | *0.378* | *0.559* | *0.256* | *0.503* | *0.155* | *0.308* | *-0.046* | 0.678* | *0.263* | *0.476* |

Table S4. Pearson correlation among soil physicochemical properties. *Correlation is significant at the 0.05 level, **significant at the 0.01 level, and ***significant at the 0.001 level.

| Items | TC | SOC | DOC | TN | AN | TP | AP | TK | AK | pH | SW | Sand | Silt |
| --- | --- | --- | --- | --- | --- | --- | --- | --- | --- | --- | --- | --- | --- |
| SOC | 0.927*** |  |  |  |  |  |  |  |  |  |  |  |  |
| DOC | 0.753** | 0.745** |  |  |  |  |  |  |  |  |  |  |  |
| TN | 0.881*** | 0.928*** | 0.793** |  |  |  |  |  |  |  |  |  |  |
| AN | 0.881*** | 0.905*** | 0.756** | 0.83*** |  |  |  |  |  |  |  |  |  |
| TP | 0.861*** | 0.818** | 0.760** | 0.800** | 0.786** |  |  |  |  |  |  |  |  |
| AP | 0.887*** | 0.828*** | 0.601* | 0.755** | 0.835*** | 0.879*** |  |  |  |  |  |  |  |
| TK | *0.221* | *0.257* | *0.112* | *0.390* | *0.249* | *0.143* | *0.340* |  |  |  |  |  |  |
| AK | 0.732** | 0.777** | 0.679* | 0.740** | 0.884*** | *0.573* | 0.658* | *0.387* |  |  |  |  |  |
| pH | -0.708* | -0.649* | *-0.318* | *-0.563* | *-0.436* | -0.731** | -0.737** | *-0.14* | *-0.156* |  |  |  |  |
| SW | 0.871*** | 0.901*** | 0.604* | 0.732** | 0.896*** | 0.641* | 0.779** | *0.119* | 0.816** | *-0.500* |  |  |  |
| Sand | -0.891*** | -0.870*** | -0.794** | -0.823** | -0.882*** | -0.858*** | -0.816** | *-0.114* | -0.842*** | *0.487* | -0.815** |  |  |
| Silt | 0.900*** | 0.893*** | 0.743** | 0.810** | 0.893*** | 0.835*** | 0.843*** | *0.152* | 0.850*** | *-0.531* | 0.859*** | -0.989*** |  |
| Clay | *0.475* | *0.392* | 0.735** | *0.550* | *0.464* | 0.627* | *0.341* | *-0.132* | *0.460* | *-0.062* | *0.253* | -0.647* | *0.528* |

Fig. S1. Alpha diversity of soil bacteria (a) and fungi (b) affected by sand deposition.

|  |  |
| --- | --- |

Fig. S2. Principal coordinate analysis (PCoA) plot of the abundances of microbial metabolism genes in the aeolian deposition farmland (a). The significant differences among the metabolism pathways were assessed by the analysis of similarities (ANOSIM) (b). Tukey's test analyzed the significant differences in the gene abundance of each metabolism pathway (c), indicated as different letters (*P* < 0.05).

| 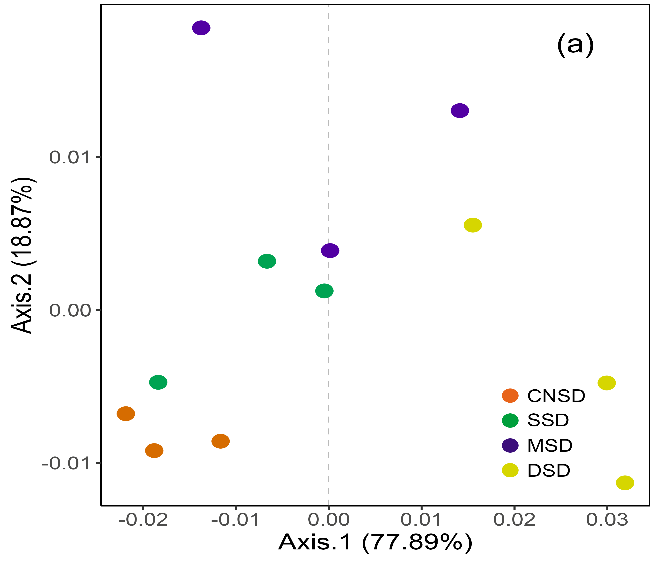 | 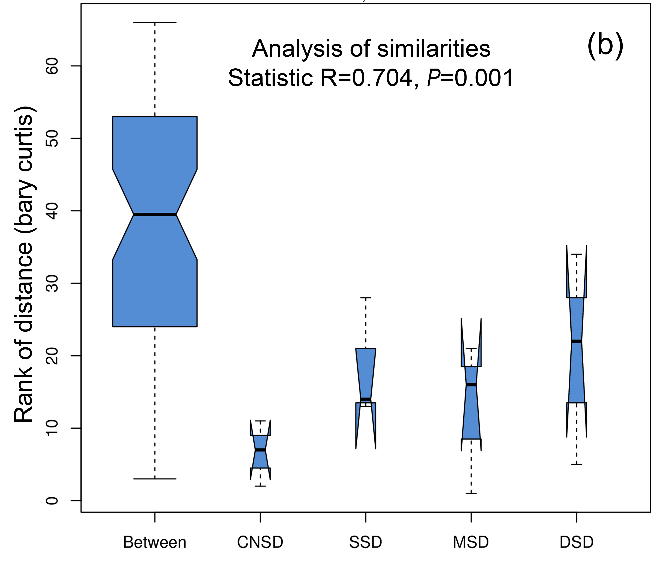 |
| --- | --- |
|  | |

Fig. S3. Principal coordinate analysis (PCoA) plot of the abundances of CAZy enzyme genes in the aeolian deposition farmland (a). The significant differences among the CAZy enzyme genes at the class level were assessed through the analysis of similarities (ANOSIM) (b), and Tukey's test analyzed the significant differences in the gene abundance of each carbohydrate-active enzyme (c), indicated as different letters (*P* < 0.05).

| 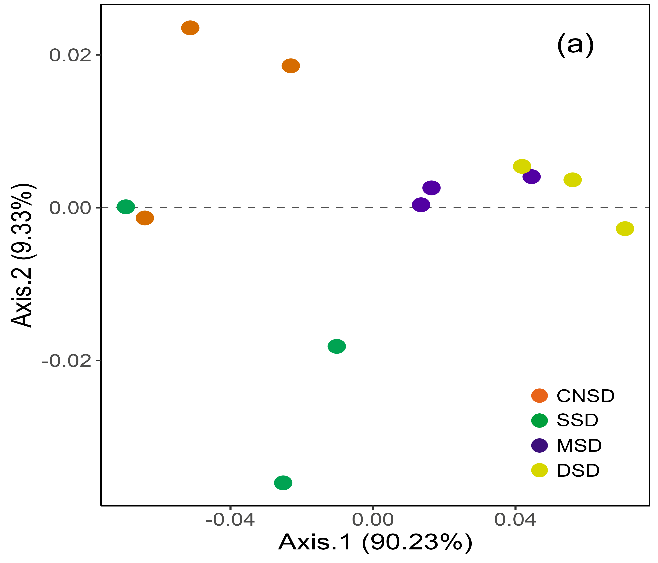 | 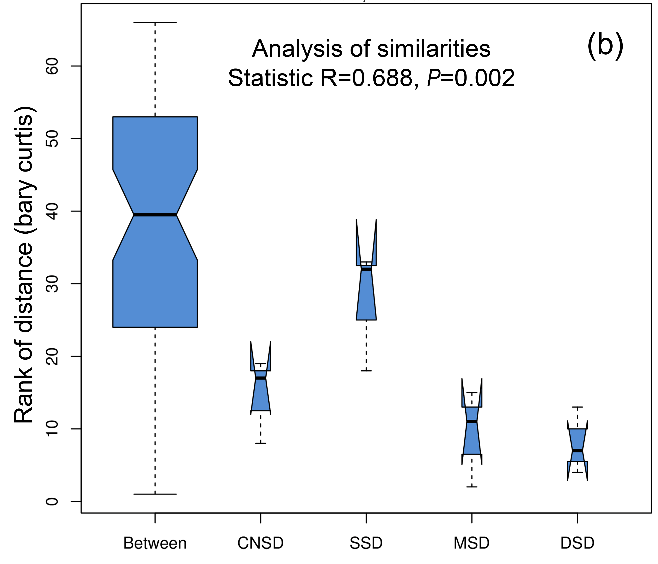 |
| --- | --- |
|  | |
